# The mutation of BCOR is highly recurrent and oncogenic in mature T-cell lymphoma

**DOI:** 10.1186/s12885-021-07806-8

**Published:** 2021-01-19

**Authors:** Jin Hyun Kang, Seung Ho Lee, Jawon Lee, Murim Choi, Junhun Cho, Seok Jin Kim, Won Seog Kim, Young Hyeh Ko, Hae Yong Yoo

**Affiliations:** 1 Clinical Research Institute, Research Institute for Future Medicine, Samsung Medical Center, Seoul, South Korea; 2 Department of Health Sciences and Technology, Samsung Advanced Institute for Health Sciences and Technology, Sungkyunkwan University, 81 Ilwon-Ro, Gangnam-Gu, Seoul, 06351 South Korea; 3 Department of Biomedical Sciences, Seoul National University College of Medicine, Seoul, South Korea; 4 Department of Pathology, Samsung Medical Center, Sungkyunkwan University School of Medicine, 81 Ilwon-Ro, Gangnam-Gu, Seoul, 06351 South Korea; 5 Samsung Biomedical Research Institute, Research Institute for Future Medicine, Samsung Medical Center, Seoul, South Korea; 6 Division of Hematology and Oncology, Department of Medicine, Samsung Medical Center, Sungkyunkwan University School of Medicine, Seoul, South Korea

**Keywords:** BCOR, T-cell lymphoma, BCL6, Gene mutation, HOX, S100 protein

## Abstract

**Background:**

BCOR acts as a corepressor of BCL6, a potent oncogenic protein in cancers of the lymphoid lineage. We have found the recurrent somatic mutation of BCOR occurred in mature T-cell lymphoma (TCL). The role of BCOR mutation in lymphoid malignancies is unknown.

**Methods:**

Lymphoma patient samples were analyzed to identify missense mutations in BCOR using Sanger sequencing. Transfection, RNA interference, immunoprecipitation, western blotting, cell proliferation, cytokine assays and quantitative real-time PCR were employed to determine the functional relevance of the novel K607E mutation in BCOR. The significant transcriptional changes were analyzed by performing DNA microarray profiling in cells expressing BCOR K607E mutant.

**Results:**

One hundred thirty-seven lymphoma patient samples were analyzed to identify K607E mutation of the BCOR gene. The BCOR K607E mutation was identified in 15 of 47 NK/T cell lymphoma cases (31.9%), 2 of 18 angioimmunoblastic T-cell lymphoma cases (11.1%), 10 of 30 peripheral T-cell lymphoma, not otherwise specified cases (33.3%), and 13 of 42 diffuse large B-cell lymphoma cases (30.9%). Molecular analysis of BCOR K607E mutation revealed that compared to the wild-type BCOR, the mutant BCOR bound to the BCL6, PCGF1, and RING1B proteins with lesser affinity. Ectopic expression of BCOR K607E mutant significantly enhanced cell proliferation, AKT phosphorylation and the expression of interleukin-2 (IL-2) with up-regulated expression of *HOX* and S100 protein genes in T cells. BCOR silencing also significantly enhanced cell proliferation, AKT phosphorylation, and IL-2 production.

**Conclusions:**

Functional analyses indicated that K607E mutation of BCOR is oncogenic in nature and can serve as a genetic marker of T-cell lymphoma.

**Supplementary Information:**

The online version contains supplementary material available at 10.1186/s12885-021-07806-8.

## Background

Malignant lymphomas can develop outside or within the lymphoid tissues, such as the lymph nodes and spleen. T-cell non-Hodgkin malignant lymphomas (NHLs) are uncommon malignancies that represent approximately 12% of all lymphomas [1]. Natural killer (NK)/T-cell lymphomas, such as extranodal NK/T-cell lymphoma nasal type (ENKTL), and peripheral T-cell lymphoma (PTCL), angioimmunoblastic T-cell lymphoma (AITL), and cutaneous T-cell lymphomas (CTCL) are rare and aggressive subtypes of T-cell NHLs. Unlike most non-Hodgkin lymphomas (which are generally B cell-related), these lymphomas are formed in response to the accumulation of one or more mutations within the T cells [2–4]. Recent genomic studies have identified highly recurrent somatic mutations in TET2, DNMT3A, IDH2, RHOA, and CD28, in diverse mature T-cell lymphoma (TCL) subtypes [5–10]. However, the roles of these mutations with respect to the regulation of T cell signaling and oncogenesis are yet to be elucidated in most cases.

BCOR (BCL-6 interacting corepressor) was identified as a corepressor that interacts selectively with the POZ domain of BCL6, a key transcription factor required for the development of germinal center B cells and diffuse large B-cell lymphomas [11–13]. BCL6 was initially identified as a potent oncoprotein in the lymphoid lineage [14, 15]. BCOR can repress transcription when tethered to a promoter and potentiate transcriptional repression by BCL-6 [11, 16]. The BCOR protein can also bind to other transcriptional factors and plays a key role in the regulation of early embryonic development and hematopoiesis [17, 18]. Additionally, BCOR forms a regulatory complex comprising ring finger protein 1B (RING1B), polycomb group ring finger 1 (PCGF1), and lysine-specific demethylase 2B (KDM2B) and functions as a component of the noncanonical polycomb repressive complex 1 (PRC1) [19, 20].

Recent whole-exome sequencing efforts have identified somatic BCOR mutations in various hematological diseases. Specifically, we and other groups reported that recurrent somatic mutations of BCOR occurred in extranodal NK/T-cell lymphoma (ENKTL) [21, 22]. However, the role of BCOR mutations in lymphoid malignancies is largely unknown. In this study, we report for the first time the frequencies of BCOR K607E mutation in various types of TCLs and the functional role of mutant BCOR in malignant lymphoma cells.

## Methods

### Validation of *BCOR* mutation by sanger sequencing

To detect BCOR mutations in lymphoma samples, genomic DNA from formalin-fixed, paraffin-embedded samples or fresh-frozen lymphoma tissues was subjected to PCR amplification using the following primers: K607E forward 5′-GAGCTTGGTGGAAGGCCGTTCTC-3′ and K607E reverse 5′- GGCACCAAAACCAGCAGGAGCTC-3′ (Additional file 1, supplementary methods). The resulting PCR products were sequenced using a nested oligonucleotide primer (5′- GGAAGGCCGTTCTCGTTTGC-3′). The QIAamp DNA Mini Kit (Qiagen) and RNeasy Mini Kit (Qiagen) were used for DNA and RNA extraction, respectively. Patient’s samples were used after obtaining informed consent from patients. The study was approved by the Institutional Review Board of Samsung Medical Center, Seoul, Korea, and was performed in accordance with the Declaration of Helsinki.

### Cloning of BCOR

A cDNA clone encoding full-length human BCOR was obtained by PCR amplification. PCR-generated DNA fragments encoding BCOR were cloned into the pcDNA3.1 vector to express Flag-tagged proteins. Mutant of BCOR, specifically Lys607Glu (AAG to GAG), was generated using the QuikChange Site-Directed Mutagenesis kit (Stratagene).

### Cell culture and transfection and RNA interference

The Jurkat (human T cell acute lymphoblastic leukemia) cell line and BJAB (Burkitt’s lymphoma) cell line were maintained in RPMI-1640, containing 10% FBS, 100 U/ml penicillin, 100 μg/ml streptomycin. To overexpress BCOR, plasmids expressing Flag-tagged wild-type BCOR and BCOR mutant (K607E) were transfected into cells using the Nucleofector I device along with Nucleofector solution V (Amaxa), according to the manufacturer’s protocols. A mixture of dsRNA nucleotides targeting different regions of BCOR mRNA and negative control small interfering RNA (scrambled siRNA) was obtained from Dharmacon. For transient expression, cells were transfected with BCOR siRNA and scrambled siRNA oligonucleotides.

### Immunoprecipitation

BCOR was purified using an anti-FLAG M2 affinity beads (Sigma) according to the manufacturer’s protocol. Briefly, cell lysates were incubated with anti-FLAG M2 beads for 4 h at 4 °C on a rotator to pull down the FLAG-tagged target protein. After incubation, immune complexes were collected by centrifugation and washed three times using ice-cold washing buffer (50 mM Tris-Cl, pH 7.4; 150 mM NaCl) at 4 °C. Whole-cell lysate and bead-bound protein complexes were separated by SDS-PAGE, followed by immunoblotting with the appropriate antibodies.

### Antibodies and immunoblotting

Cells were lysed in RIPA buffer. Cell lysate samples were resolved by SDS-PAGE and blotted to PVDF membranes. The blots were probed with anti- FLAG (Sigma), anti-BCOR (Bethyl Laboratoris), anti-BCL6 (Cell Signaling), anti-PCGF1 (Abcam), anti-RING1B (Cell Signaling), and anti-phospho-AKT (Cell Signaling), followed by anti-rabbit HRP-conjugated antibody (Bio-Rad). Immunostained proteins were detected by ECL (Amersham Pharmacia Biotech). Additional details are provided in supplementary methods (Additional file 1).

### Cell proliferation and cytokine assays

Cell proliferation and cytokine assays were determined by using a CCK8 assay kit and ELISA kit. Additional details are provided in supplementary methods (Additional file 1).

### Gene expression analysis

Gene expression analysis using Agilent’s Gene Expression Hybridization Kit (GPL13497) was performed for cell lines expressing wild-type BCOR and BCOR K607E mutant, as well as for cell lines transfected with BCOR siRNA. Differentially expressed genes were selected by performing Student’s t-test using normalized expression counts. HOX and S100 were selected and extracted from the microarray data and their expression visualized using R (version 3.6.1). Finally, gene ontology analysis was performed using the ToppGene suite.

### RNA isolation, reverse transcription reaction, and quantitative real-time PCR

Total mRNA was extracted from cultured cells or fresh-frozen lymphoma tissues using TRIzol reagent (Ambion by Life Technologies). First-strand cDNA was synthesized from 2 μg total RNA using SuperScript II RNase Reverse Transcriptase (Invitrogen). Quantitative real-time PCR was carried out with SYBR Green Master Mix (Applied Biosystems) using the Applied Biosystems QuantStudio™ 6 Flex Real-Time PCR Instrument (384-well). Relative expression was evaluated using the comparative cycle threshold (2^-△△Ct^) method.

### Statistical analysis

All data were analyzed by independent *t*-tests or analysis of variance using SPSS software. Differences with a *p*-value < 0.05 were considered statistically significant. Results are expressed as the mean ± standard error of the mean (SEM).

## Results

### Frequent alterations in the *BCOR* gene

**Fig. 1:**
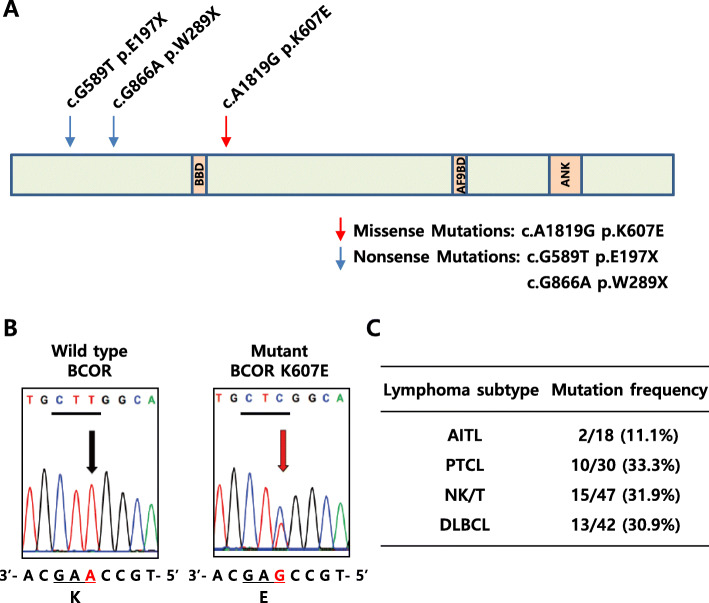
Validation of K607E mutation on BCOR. **a** Schematic representation of mutation position and functional domains of the BCOR protein. **b** Representative sequencing traces of wild-type and K607E mutant BCOR. Lysine (AAG) was changed to Glutamic acid (GAG) at the 607th amino acid of BCOR. Left panel shows sequence obtained from the wild-type genomic DNA. Right panel shows sequence obtained from an NK/T-cell lymphoma sample. Arrows and red letters denote the location of the base change. The mutant sequence trace shows heterozygosity. **c** The genomic DNA of BCOR from lymphoma patients was analyzed by PCR amplification and Sanger sequencing. BBD, BCL6 Binding Domain; AF9BD, AF9 Binding Domain; ANK, Ankyrin Repeats

We have previously reported that recurrent somatic mutations of BCOR occurred in lymphoid malignancies, particularly extranodal NK/T-cell lymphoma (ENKTL) [21]. In order to more precisely estimate the frequency of BCOR mutation in lymphoma patients, we examined genomic DNA from 47 NK/T cell lymphoma patient samples and detected two types of nonsense mutations (E197X and W289X) along with one type of missense mutation (K607E) (Fig. 1a, b and Figure S1, Additional file 2). In contrast to two nonsense mutations in BCOR, *BCOR 1819 A > G* (K607E substitution) was found at a high frequency in 15 of 47 NK/T cell lymphoma patients (31.9%).

To better estimate the K607E mutation rate and to determine subtype specificity among diverse categories of lymphoma, we expanded the patient cohort to include an additional 18 angioimmunoblastic T-cell lymphoma (AITL) cases, 30 peripheral T-cell lymphoma (PTCL) cases for T-cell lymphoma, and 42 diffuse large B-cell lymphoma (DLBCL) cases for B-cell lymphoma (Fig. 1c). The K607E mutation of BCOR was detected in 2 of 18 AITL (11.1%), 10 of 30 PTCL (33.3%), and 13 of 42 DLBCL (30.9%). All mutation-positive cases were heterozygotes, as validated by Sanger sequencing of the PCR products. The results indicated that K607E mutation of BCOR occurs in all tested subtypes of lymphoma with an overall frequency of 30%.

### Functional analyses of K607E mutation in BCOR

**Fig. 2:**
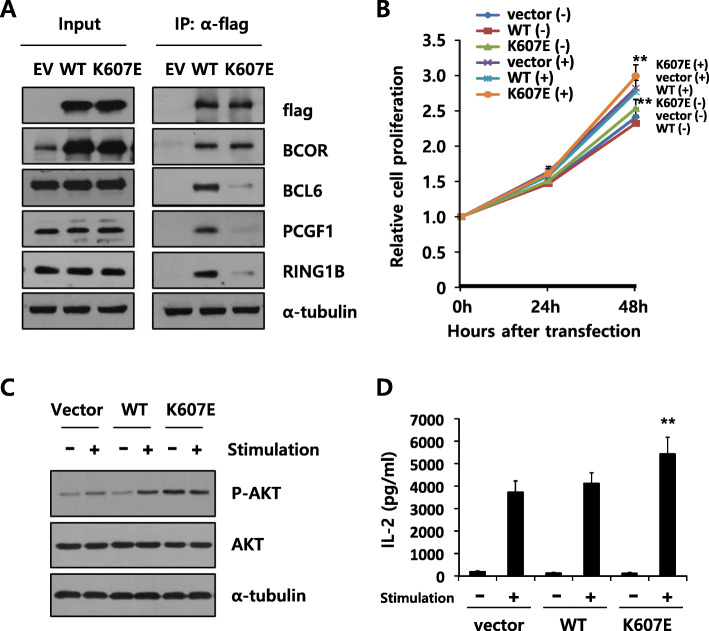
Functional analyses of the K607E mutation in BCOR. **a** Expression of BCOR K607E mutant decreased the interaction with BCL6, PCGF1, and RING1B. BJAB cells were transfected with plasmids encoding flag-tagged wild-type (WT) BCOR or K607E mutant, and immunoprecipitation was performed using an anti-flag M2 antibody. Immunoblotting analysis was performed using anti-BCL6, PCGF1, and RING1B. Alpha-tubulin was used as the loading control. This result is representative of four independent experiments. The uncropped blots are presented in Supplementary Figure S4 (Additional file 5). **b** Enhanced cell proliferation in K607E mutant expressing mutated BCOR (**P < 0.01 compared with cells expressing wild-type BCOR). Jurkat cells were transfected with wild-type BCOR or K607E mutant expressing plasmids. After 48 h, cells were stimulated with plate-bound anti-CD3/CD28. Data are shown as the mean ± SEM of six independent experiments performed in triplicates. **c** Expression of BCOR K607E mutant enhanced phosphorylation of AKT without stimulation. The blot was probed with anti–phospho-AKT antibody, and subsequently stripped and reprobed with anti-AKT antibody. Alpha-tubulin was used as the loading control. This result is representative of three independent experiments. The uncropped blots are presented in Supplementary Figure S4 (Additional file 5). **d** Expression of BCOR K607E mutant enhanced IL-2 production after stimulation with PMA and ionomycin (**P < 0.01 compared with cells expressing wild-type BCOR). The results are expressed as the mean ± SEM of six independent experiments performed in triplicates

We next explored the functional significance of K607E mutation in BCOR. The K607E mutation is located near the BCL6 binding domain (Fig. 1a), suggesting that the mutation may affect the interaction of BCOR with the partner proteins. To analyze the effect of BCOR K607E mutation on BCL6 binding, we transfected plasmids encoding Flag-tagged wild-type BCOR or BCOR K607E mutant into BJAB cells. The interaction of BCL6 with the BCOR K607E mutant decreased compared to that observed with the wild-type BCOR (Fig. 2a). Furthermore, the binding of PCGF1 and RING1B, components of the BCOR complex to BCOR K607E mutant also decreased. These results indicate that the substitution of 607th amino acid from lysine to glutamic acid in BCOR changes the binding of multiple partner proteins.

We subsequently analyzed the effect of the BCOR K607E mutation on cell proliferation and AKT phosphorylation. Jurkat (human T-cell acute lymphoblastic leukemia) and Hut-78 (human cutaneous T-cell lymphoma) cells transfected with the construct expressing BCOR K607E showed an approximately 20% higher proliferation rate than cells expressing wild-type BCOR, either with or without co-stimulation with anti-CD3 and anti-CD28 (Fig. 2b and Figure S2A, Additional file 3). This result indicates that the BCOR K607E mutation resulted in constitutive activation of T-cell stimulation and cell proliferation. We also examined the phosphorylation of AKT, which is involved in T cell signaling pathways and cell proliferation [23]. As expected, anti-CD3/CD28 co-stimulation resulted in increased AKT phosphorylation (Fig. 2c). Importantly, cells expressing BCOR K607E showed a higher level of AKT phosphorylation compared to cells expressing wild-type BCOR even without stimulation. These results are consistent with the finding that mutant BCOR results in constitutive activation of signaling.

**Fig. 3:**
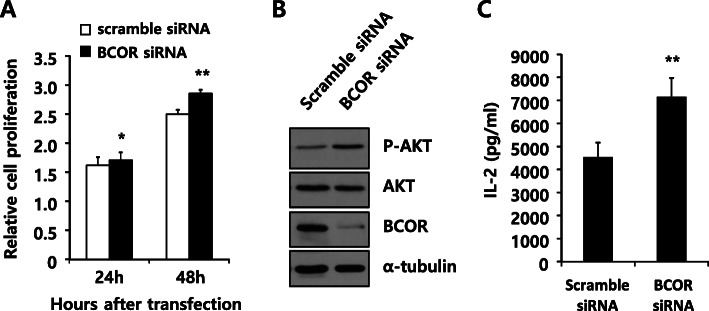
Silencing of BCOR increased cell proliferation, AKT phosphorylation, and IL-2 production. Jurkat cells were transfected with scrambled siRNA or BCOR siRNA oligonucleotides. **a** After transfection, relative cell proliferation was determined using a cell counting kit (CCK-8) for the indicated time. Data are shown as the mean ± SEM of five independent experiments performed in triplicates (**P < 0.01 compared with cells transfected with scrambled siRNA). **b** Cell lysates (20 μg) were prepared 48 h after transfection and processed for immunoblotting with the indicated antibodies. Alpha-tubulin was used as the loading control. This result is representative of three independent experiments. The uncropped blots are shown in Supplementary Figure S5 (Additional file 6). **c** Analysis of the production of IL-2 by ELISA. After transfection, cells were stimulated with PMA and ionomycin. Data are shown as the mean ± SEM of five independent experiments performed in triplicates (**P < 0.01 compared with cells transfected with scrambled siRNA)

We also analyzed the effect of BCOR K607E mutation on cytokine production by T cells (Fig. 2d and Figure S2B, Additional file 3). Expression of BCOR K607E mutant in T cells enhanced the production of IL-2, a key cytokine for T-cell activation and proliferation [24], compared to that of wild-type BCOR after co-stimulation with PMA and ionomycin. Similar to the results obtained upon expressing BCOR K607E, BCOR silencing also significantly enhanced cell proliferation, AKT phosphorylation, as well as IL-2 production (Fig. 3). Collectively, these results indicate that the K607E mutation of BCOR is likely a loss-of-function mutation.

### Gene expression signature of BCOR K607E mutant in T-cell lymphoma

**Fig. 4:**
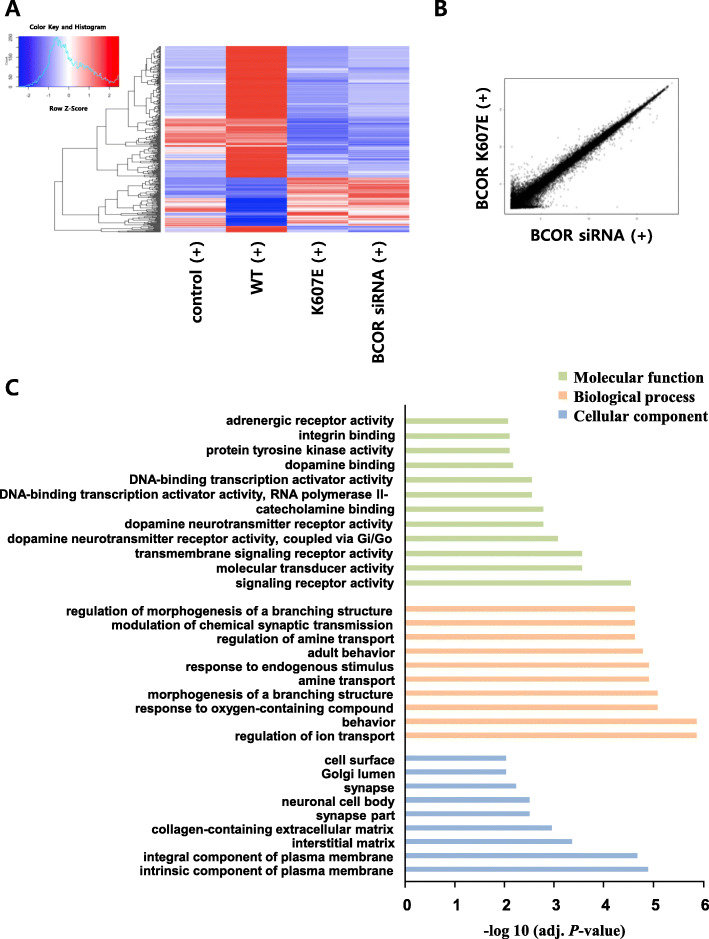
Gene expression profiles of BCOR mutant expressing cells. **a** Heat map of genes with 1.5-fold as well as *P* < 0.05 mRNA expression differences between wild-type BCOR and BCOR K607E mutant expressing cells stimulated with PMA and ionomycin. Ninety genes were up-regulated and 256 genes were down-regulated. **b** Scatterplot representation of correlation between cells expressing the BCOR K607E mutant and transfected BCOR siRNA. **c** Molecular functions, biological processes, and cellular components that were upregulated in the BCOR K607E mutant expressing cells as compared to cells expressing the wild-type BCOR, determined via GO analysis of genes

The BCOR protein acts as a corepressor of BCL6 and can also bind to other transcriptional factors [11, 17]. Binding assays showed that the binding of several partner proteins with BCOR K607E mutant decreased compared to the level seen with wild-type BCOR (Fig. 2a). To identify the significant transcriptional changes induced by BCOR K607E mutant in T cells, we compared the gene expression profiles in cells expressing wild-type BCOR, cells expressing BCOR K607E mutant and cells with BCOR knocking-down using DNA microarray profiling (Fig. 4). Using a filter criterion of at least a 1.5-fold change with *p* < 0.05, the number of genes with altered expression in Jurkat cells expressing BCOR K607E mutant and those expressing wild-type BCOR were determined. It was observed that the expression increased for 90 genes and decreased for 256 genes in cells expressing BCOR K607E mutant (Fig. 4a). Furthermore, the mutant samples clustered more similarly with the siRNA knockdown samples than with the wild-type sample (Fig. 4a and b), suggesting that BCOR K607E is a loss-of-function mutant. Gene ontology analysis revealed that gene clusters enriched in the genes upregulated by BCOR K607E mutant were associated with signaling receptor activity, molecular transducer activity, regulation of ion transport and behavior, as well as intrinsic and integral components of plasma membrane related genes (Fig. 4c).

Next, we focused on genes upregulated in BCOR K607E mutant expressing cells compared to wild-type BCOR expressing cells. The genes with significant changes in expression are listed in Table 1. We also performed quantitative real-time PCR (qRT–PCR) for checking the expression of select genes (*ISPD, HOXB6, ATP13A4, MAK,* and *SLC7A8*) from among the list of top 10 genes upregulated by BCOR K607E mutant (Figure S3A, Additional file 4), and these findings were consistent with the gene expression profiling results.

**Table 1:** Top 10 up-regulated and down-regulated genes by BCOR K607E mutant

No	Gene Symbol	Gene Title	fold change	
			K607E/WT	siRNA/WT
**Top 10 up-regulated genes**				
1	ISPD	isoprenoid synthase domain containing	2.88	1.74
2	ESRG	embryonic stem cell related (non-protein coding)	2.62	1.83
3	HOXB6	homeobox B6	2.50	2.01
4	LOC101929560	uncharacterized LOC101929560	2.48	1.62
5	CNKSR3	CNKSR family member 3	2.40	1.55
6	ATP13A4	ATPase type 13A4	2.31	1.73
7	MAK	male germ cell-associated kinase	2.28	1.60
8	SLC7A8	solute carrier family 7 (amino acid transporter light chain, L system), member 8	2.27	1.83
9	PTAFR	platelet-activating factor receptor	2.24	1.90
10	LOC101927250	uncharacterized LOC101927250	2.23	1.83
**Top 10 down-regulated genes**				
1	DNMBP-AS1	DNMBP antisense RNA 1	0.27	0.30
2	DNAJB8-AS1	DNAJB8 antisense RNA 1	0.29	0.32
3	GRIN1	glutamate receptor, ionotropic, N-methyl D-aspartate 1	0.32	0.35
4	SORBS2	sorbin and SH3 domain containing 2	0.33	0.34
5	NTM	neurotrimin	0.33	0.36
6	ARRDC3-AS1	ARRDC3 antisense RNA 1	0.33	0.37
7	ARHGAP28	Rho GTPase activating protein 28	0.34	0.38
8	MAGEA11	melanoma antigen family A, 11	0.34	0.37
9	LOC93432	maltase-glucoamylase (alpha-glucosidase)	0.36	0.39
10	lnc-OBFC2A-3	lnc-OBFC2A-3:1	0.36	0.41

**Fig. 5:**
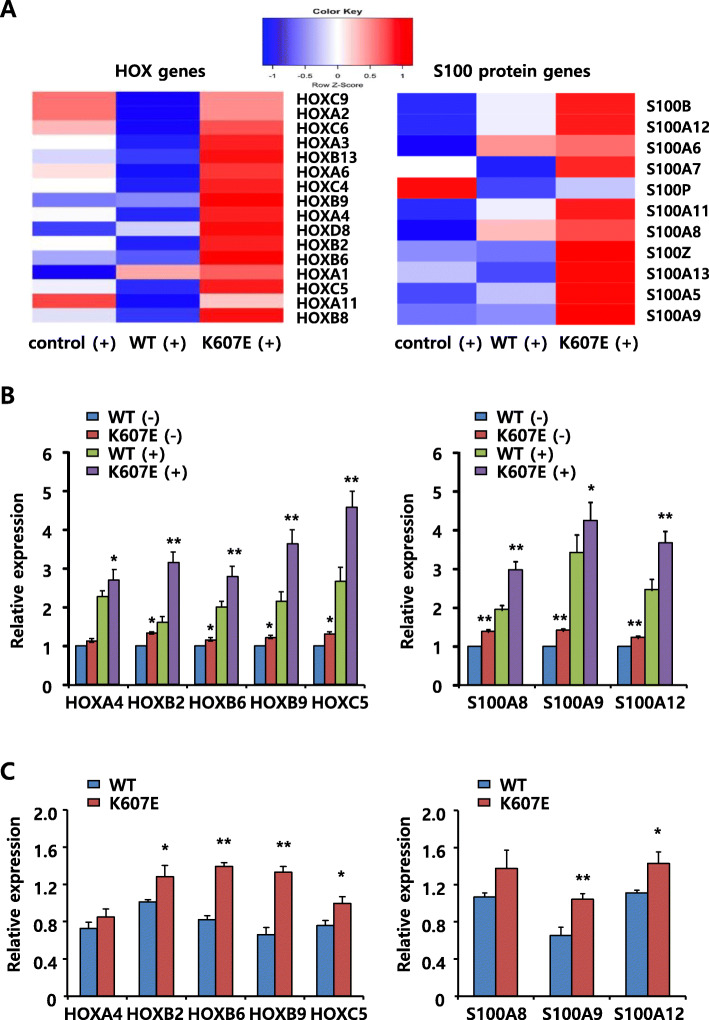
BCOR regulated HOX cluster genes and S100A protein genes. Jurkat cells were transfected with wild-type BCOR or BCOR K607E mutant expressing plasmids, and scrambled siRNA or BCOR siRNA. After 48 h, cells were stimulated with PMA and ionomycin for 6 h for analysis of gene expression. **a** Hierarchical cluster analysis of HOX and S100A protein expression in wild-type BCOR or BCOR K607E mutant expressing cells revealed the upregulation of several *HOX* genes and S100A protein genes by BCOR K607E mutant. **b** and **c** Quantitative PCR validation of differentially expressed genes selected from the microarray analysis between wild-type BCOR and BCOR K607E mutant expressing cells (**b**) and fresh-frozen tumor samples (**c**). GAPDH was used as a control to normalize the levels of these transcripts. Data are shown as the mean ± SEM of five independent experiments performed in triplicates (*P < 0.05, **P < 0.01 compared with cells transfected with wild-type BCOR expressing plasmids and wild-type BCOR tumor samples)

To date, numerous studies have shown that functional abnormalities of HOX transcription factors play a critical role in the development and progression of many types of cancers [25, 26]. *HOXB6* was identified as the 3rd most upregulated gene in the highly expressed gene list (Top 10 upregulated genes, Table 1). One of the known BCL6 targets, *S100A11* [27] was moderately upregulated in cells expressing the K607E mutant as well as in cells transfected with the BCOR siRNA. We further performed hierarchical cluster analysis of HOX genes (*n* = 39) and S100 protein genes (*n* = 19) present in the differentially expressed genes set. These HOX and S100 protein genes showed the greatest fold changes in cells expressing the BCOR mutant (Fig. 5a). In order to verify the microarray results, qRT-PCR was used to check the expression of select genes (Fig. 5b). BCOR K607E mutant-expressing cells exhibited significantly upregulated HOX genes (*HOXA4, HOXB2, HOXB6, HOXB9* and *HOXC5*) and S100 protein (*S100A8, S100A9*, and *S100A12*) genes expression by approximately 1.3–2.5 fold compared with wild-type BCOR-expressing cells. We also checked the expression of selected genes among genes upregulated by BCOR K607E mutant in fresh-frozen tumor patient samples by qRT-PCR. These genes (*HOX, S100 protein, ISPD, ATP13A4, MAK*, and *SLC7A8*) were also significantly upregulated in tumor samples expressing BCOR K607E mutant as compared to that in tumor samples expressing wild-type BCOR (Fig. 5c and Figure S3B, Additional file 4), and these findings were consistent with gene expression profiling results obtained in cell lines. Overall, these results indicated that BCOR mutants can activate the expression of several transcription factors, such as HOX and S100 protein.

## Discussion

Recurrent inactivating somatic BCOR mutations have been identified in various hematological malignancies, acute myeloid leukemia (AML), myelodysplastic syndrome (MDS), chronic myelomonocytic leukemia, medulloblastoma, and retinoblastoma [28, 29]. We and other groups have previously reported recurrent somatic mutations of BCOR in T cell lymphoid malignancies [21, 22]. However, the role of BCOR mutations in the regulation of T-cell signaling and oncogenesis remains to be elucidated. In this study, we detected two cases with nonsense mutations, E197X (26.1%) and W289X (5.9%), and one case with a missense mutation, K607E (31.9%) in 47 NK/T-cell lymphoma. We further identified a novel K607E mutation of BCOR gene in a cohort of 137 lymphoma patients (Fig. 1). Clinically, BCOR K607E mutation in lymphoma patients has no correlation with gender, age, lesion location, and survival.

Recently, the tumor suppressor function of BCOR has been confirmed in vivo in a Myc-driven lymphomagenesis model as well as in transgenic mice expressing a truncated form of BCOR (partial internal deletion) that cannot bind to BCL6 [30, 31]. However, its tumor suppressor function remains largely uncharacterized. Our data showed that the K607E mutation in BCOR—which results in the protein being unable to bind to BCL6— significantly enhanced cell proliferation, AKT phosphorylation, and IL-2 production in T cell lymphoma lines (Fig. 2). The effects of BCOR K607E mutant expression on cells were similar to those of BCOR silencing, rather than to those of BCOR wild-type overexpression (Fig. 3). In both the BCOR K607E mutant-expressing and BCOR-silenced groups, gene upregulation was highly correlated, as compared with the wild-type BCOR (Fig. 4 and Table 1). These results provide evidenced that BCOR K607E mutation possesses a tumor activator role, suggesting that BCOR potentially functions as a tumor suppressor in T cell lymphoma.

BCL6 was initially discovered as an oncogene in B-cell lymphomas and consequently emerged as a therapeutic target [32–34]. Furthermore, BCL6 is also expressed in the malignant T-cells of AITL, anaplastic large cell lymphoma (ALCL), and follicular helper T cells (Tfh cells) [35–37]. BCL6-driven gene expression in T-cells is less well characterized. BCOR is likely to be a crucial mediator of BCL6 function in these cancers, whereas BCL6 can coordinate the actions of the BCOR polycomb-like complex (BCOR, PCGF1, RING1B, and KDM2B) to potently repress target genes [11]. As shown in Fig. 5, the expression of HOX (*HOXA4, HOXB2, HOXB6, HOXB9* and *HOXC5*) and S100 protein (*S100A8, S100A9* and *S100A12*) genes was significantly and markedly higher in BCOR mutant-expressing T cells than that in BCOR wild-type-expressing T cells. Overall, these results indicate that BCOR mutation can activate the expression of HOX and S100 proteins.

The homeodomain genes (*HOX*) encode a family of highly conserved transcription factors that play an important role in embryonic development, hematopoiesis, and leukemogenesis [25, 26]. Elevated HOX gene expression has been observed in acute myeloid leukemia (AML) and is correlated with poor prognosis [38, 39]. HOXB6 is included in the list of highly expressed genes in this study. We also examined HOX gene expression patterns in T cells expressing BCOR K607E or wild-type BCOR by qRT-PCR (Fig. 5). It was observed that BCOR K607E mutant significantly upregulated the expression of particular HOX genes. Interestingly, we also discovered an apparent association between PCGF1 and HOX expression in the group of T cells expressing BCOR K607E. Polycomb-group (PcG) proteins are known to suppress the expression of HOX genes, and a recent study showed that polycomb group ring finger 1 (PCGF1), a member of the BCOR complex, is involved in the regulation of HOX gene expression [40]. In this study, the K607E mutant showed decreased binding to PCGF1 and RING1B, indicating that the K607E mutant specifically abrogated the HOX repressor function of PCGF1.

S100 proteins comprise a group of damage-associated molecular pattern (DAMP) molecules considered to be important inflammatory mediators [41]. Elevated levels of S100 proteins has been detected in inflammation, neoplastic tumor cells, and various human cancers [42–44]. In particular, S100A8, S100A9 and S100A12 are highly abundant proteins released by neutrophils and have been identified as important biomarkers in many inflammatory diseases and cancers [41]. However, the role of these specific S100 proteins in the pathogenesis of such diseases is entirely unknown. In this study, we showed that the transcripts of three S100 proteins S100A8, S100A9 and S100A12 were upregulated in BCOR K607E mutant expressing cells compared with wild-type BCOR expressing cells (Fig. 5). We thus propose that they serve as important mediators in the molecular pathogenesis of T cell lymphoma.

## Conclusions

In summary, we identified and functional characterized the K607E mutation of BCOR in T cell lymphoma. We also reported the gene expression profile of T lymphoma cell lines expressing BCOR K607E mutant, and suggested several novel target genes involved in the pathogenesis of this disease. Hence, we propose that BCOR plays the role of a tumor suppressor in the pathogenesis of T lymphocyte malignancies. The K607E mutation of BCOR is oncogenic in nature and can serve as a genetic marker for T-cell lymphoma.

## Supplementary Information

**Additional file 1:** **Supplementary Methods**.

**Additional file 2:** **Figure S1.** Validation of K607E mutation on BCOR in FFPE tissues and fresh frozen tissues from the same patients. Representative sequencing traces of wild-type and K607E mutant BCOR. Lysine (AAG) was changed to Glutamic acid (GAG) at the 607th amino acid of BCOR. Arrows and red letters denote the location of the base change (#1–5 wild-type BCOR samples and #6–10 BCOR K607E mutant samples).

**Additional file 3:** **Figure S2.** Expression of BCOR K607E mutant enhanced cell proliferation and IL- 2 production. **a** Hut78 cells were transfected with wild-type BCOR or K607E mutant expressing plasmids. After 48 h, cells were stimulated with plate-bound anti-CD3/CD28. After stimulation, cell proliferation was determined using a cell counting kit (CCK-8). Data are shown as the mean ± SEM of seven independent experiments performed in triplicate (**P < 0.01 compared with cells expressing wild-type BCOR). **b** After transfection, Hut78 cells were stimulated with PMA and ionomycin. The concentrations of IL-2 were estimated by ELISA. Data are shown as the mean ± SEM of five independent experiments performed in triplicate (**P < 0.01 compared with cells expressing wild-type BCOR).

**Additional file 4:** **Figure S3.** HOXB6, ISPD, ATP13A4, MAK, and SLC7A8 were up-regulated in BCOR K607E mutant expressing cells (a) and BCOR K607E mutant tumor samples (b). Quantitative PCR analysis of the indicated genes from the list of Top10 genes showed upregulation by BCOR K607E mutant. GAPDH was used as a control to normalize the levels of these transcripts. Data are shown as the mean ± SEM of six independent experiments performed in triplicates (*P < 0.05, **P < 0.01 compared with cells transfected with wild-type BCOR and wild-type BCOR tumor samples).

**Additional file 5:** **Figure S4.** The uncropped blots of Fig. 2a and c are displayed.

**Additional file 6:** **Figure S5.** The uncropped blots of Fig. 3b are displayed.

## Data Availability

All data generated or analyzed during this study are available from the corresponding author upon reasonable request.

## Abbreviations

AITL: Angioimmunoblastic T-cell lymphoma
AML: Acute myeloid leukemia
ALCL: Anaplastic large cell lymphoma
CTCL: Cutaneous T-cell lymphomas
DLBCL: Diffuse large B-cell lymphoma
DAMP: Damage-associated molecular pattern
ENKTL: Extranodal NK/T-cell lymphoma
IL-2: Interleukin-2
KDM2B: Lysine-specific demethylase 2B
MDS: Myelodysplastic syndrome
NHLs: Non-Hodgkin malignant lymphomas
PTCL: Peripheral T-cell lymphoma
PcG: Polycomb-group
PCGF1: Polycomb group ring finger 1
PRC1: Polycomb repressive complex 1
qRT–PCR: Quantitative real-time PCR
RING1B: Ring finger protein 1B
TCL: T cell lymphoma
Tfh cells: Follicular helper T-cells

## References

[1] A clinical evaluation of the International Lymphoma Study Group classification of non-Hodgkin's lymphoma (1997). The Non-Hodgkin's Lymphoma Classification Project. Blood..

[2] Tse E, Kwong Y-L. The diagnosis and management of NK/T-cell lymphomas. J Hematol Oncol. 2017;10(1):85. 10.1186/s13045-017-0452-9 PMC5391564 28410601

[3] Moskowitz AJ, Lunning MA, Horwitz SM (2014). How I treat the peripheral T-cell lymphomas. Blood..

[4] Vose J, Armitage J, Weisenburger D (2008). International peripheral T-cell and natural killer/T-cell lymphoma study: pathology findings and clinical outcomes. J Clin Oncol.

[5] Cairns RA, Iqbal J, Lemonnier F, Kucuk C, de Leval L, Jais JP (2012). IDH2 mutations are frequent in angioimmunoblastic T-cell lymphoma. Blood..

[6] Lemonnier F, Couronne L, Parrens M, Jais JP, Travert M, Lamant L (2012). Recurrent TET2 mutations in peripheral T-cell lymphomas correlate with TFH-like features and adverse clinical parameters. Blood..

[7] Palomero T, Couronne L, Khiabanian H, Kim MY, Ambesi-Impiombato A, Perez-Garcia A (2014). Recurrent mutations in epigenetic regulators, RHOA and FYN kinase in peripheral T cell lymphomas. Nat Genet.

[8] Sakata-Yanagimoto M, Enami T, Yoshida K, Shiraishi Y, Ishii R, Miyake Y (2014). Somatic RHOA mutation in angioimmunoblastic T cell lymphoma. Nat Genet.

[9] Yoo HY, Sung MK, Lee SH, Kim S, Lee H, Park S (2014). A recurrent inactivating mutation in RHOA GTPase in angioimmunoblastic T cell lymphoma. Nat Genet.

[10] Lee SH, Kim JS, Kim J, Kim SJ, Kim WS, Lee S (2015). A highly recurrent novel missense mutation in CD28 among angioimmunoblastic T-cell lymphoma patients. Haematologica..

[11] Huynh KD, Fischle W, Verdin E, Bardwell VJ (2000). BCoR, a novel corepressor involved in BCL-6 repression. Genes Dev.

[12] Klein U, Dalla-Favera R (2008). Germinal centres: role in B-cell physiology and malignancy. Nat Rev Immunol.

[13] Cattoretti G, Pasqualucci L, Ballon G, Tam W, Nandula SV, Shen Q (2005). Deregulated BCL6 expression recapitulates the pathogenesis of human diffuse large B cell lymphomas in mice. Cancer Cell.

[14] Jardin F, Ruminy P, Bastard C, Tilly H (2007). The BCL6 proto-oncogene: a leading role during germinal center development and lymphomagenesis. Pathol Biol (Paris).

[15] Bunting KL, Melnick AM (2013). New effector functions and regulatory mechanisms of BCL6 in normal and malignant lymphocytes. Curr Opin Immunol.

[16] Chang CC, Ye BH, Chaganti RS, Dalla-Favera R (1996). BCL-6, a POZ/zinc-finger protein, is a sequence-specific transcriptional repressor. Proc Natl Acad Sci U S A.

[17] Tiacci E, Grossmann V, Martelli MP, Kohlmann A, Haferlach T, Falini B (2012). The corepressors BCOR and BCORL1: two novel players in acute myeloid leukemia. Haematologica..

[18] Wamstad JA, Corcoran CM, Keating AM, Bardwell VJ (2008). Role of the transcriptional corepressor Bcor in embryonic stem cell differentiation and early embryonic development. PLoS One.

[19] Gearhart MD, Corcoran CM, Wamstad JA, Bardwell VJ (2006). Polycomb group and SCF ubiquitin ligases are found in a novel BCOR complex that is recruited to BCL6 targets. Mol Cell Biol.

[20] Yamamoto Y, Abe A, Emi N (2014). Clarifying the impact of polycomb complex component disruption in human cancers. Mol Cancer Res.

[21] Lee S, Park HY, Kang SY, Kim SJ, Hwang J, Lee S (2015). Genetic alterations of JAK/STAT cascade and histone modification in extranodal NK/T-cell lymphoma nasal type. Oncotarget..

[22] Dobashi A, Tsuyama N, Asaka R, Togashi Y, Ueda K, Sakata S (2016). Frequent BCOR aberrations in extranodal NK/T-cell lymphoma, nasal type. Genes Chromosomes Cancer.

[23] Brazil DP, Park J, Hemmings BA (2002). PKB binding proteins. Getting in on the Akt. Cell.

[24] Toribio ML, Gutierrez-Ramos JC, Pezzi L, Marcos MA, Martinez C (1989). Interleukin-2-dependent autocrine proliferation in T-cell development. Nature..

[25] Shah N, Sukumar S (2010). The Hox genes and their roles in oncogenesis. Nat Rev Cancer.

[26] Luo Z, Rhie SK, Farnham PJ. The enigmatic HOX genes: can we crack their code? Cancers (Basel). 2019;11(3):323. 10.3390/cancers11030323 PMC6468460 30866492

[27] Sawant DV, Sehra S, Nguyen ET, Jadhav R, Englert K, Shinnakasu R (2012). Bcl6 controls the Th2 inflammatory activity of regulatory T cells by repressing Gata3 function. J Immunol.

[28] Grossmann V, Tiacci E, Holmes AB, Kohlmann A, Martelli MP, Kern W (2011). Whole-exome sequencing identifies somatic mutations of BCOR in acute myeloid leukemia with normal karyotype. Blood..

[29] Damm F, Chesnais V, Nagata Y, Yoshida K, Scourzic L, Okuno Y (2013). BCOR and BCORL1 mutations in myelodysplastic syndromes and related disorders. Blood..

[30] Lefebure M, Tothill RW, Kruse E, Hawkins ED, Shortt J, Matthews GM (2017). Genomic characterisation of Emu-Myc mouse lymphomas identifies Bcor as a Myc co-operative tumour-suppressor gene. Nat Commun.

[31] Tanaka T, Nakajima-Takagi Y, Aoyama K, Tara S, Oshima M, Saraya A (2017). Internal deletion of BCOR reveals a tumor suppressor function for BCOR in T lymphocyte malignancies. J Exp Med.

[32] Cardenas MG, Oswald E, Yu W, Xue F, MacKerell AD, Melnick AM (2017). The expanding role of the BCL6 oncoprotein as a cancer therapeutic target. Clin Cancer Res.

[33] Ye BH, Lista F, Lo Coco F, Knowles DM, Offit K, Chaganti RS (1993). Alterations of a zinc finger-encoding gene, BCL-6, in diffuse large-cell lymphoma. Science..

[34] Ye BH, Cattoretti G, Shen Q, Zhang J, Hawe N, de Waard R (1997). The BCL-6 proto-oncogene controls germinal-centre formation and Th2-type inflammation. Nat Genet.

[35] Lamant L, de Reynies A, Duplantier MM, Rickman DS, Sabourdy F, Giuriato S (2007). Gene-expression profiling of systemic anaplastic large-cell lymphoma reveals differences based on ALK status and two distinct morphologic ALK+ subtypes. Blood..

[36] Piccaluga PP, Agostinelli C, Califano A, Carbone A, Fantoni L, Ferrari S (2007). Gene expression analysis of angioimmunoblastic lymphoma indicates derivation from T follicular helper cells and vascular endothelial growth factor deregulation. Cancer Res.

[37] Choi YS, Yang JA, Crotty S (2013). Dynamic regulation of Bcl6 in follicular helper CD4 T (Tfh) cells. Curr Opin Immunol.

[38] Kawagoe H, Humphries RK, Blair A, Sutherland HJ, Hogge DE (1999). Expression of HOX genes, HOX cofactors, and MLL in phenotypically and functionally defined subpopulations of leukemic and normal human hematopoietic cells. Leukemia..

[39] Andreeff M, Ruvolo V, Gadgil S, Zeng C, Coombes K, Chen W (2008). HOX expression patterns identify a common signature for favorable AML. Leukemia..

[40] Ross K, Sedello AK, Todd GP, Paszkowski-Rogacz M, Bird AW, Ding L (2012). Polycomb group ring finger 1 cooperates with Runx1 in regulating differentiation and self-renewal of hematopoietic cells. Blood..

[41] Foell D, Wittkowski H, Vogl T, Roth J (2007). S100 proteins expressed in phagocytes: a novel group of damage-associated molecular pattern molecules. J Leukoc Biol.

[42] Bresnick AR, Weber DJ, Zimmer DB (2015). S100 proteins in cancer. Nat Rev Cancer.

[43] Brenner AK, Bruserud O (2018). S100 proteins in acute myeloid leukemia. Neoplasia..

[44] Shabani F, Farasat A, Mahdavi M, Gheibi N (2018). Calprotectin (S100A8/S100A9): a key protein between inflammation and cancer. Inflamm Res.

