# Supplementary material for: The mutation of BCOR is highly recurrent and oncogenic in mature T-cell lymphoma
Source: BMC Cancer. 2021 Jan 19;21:82. doi: 10.1186/s12885-021-07806-8 (PMC7816311; doi:10.1186/s12885-021-07806-8)
Supplement: Supplementary file 1 — Additional file 1: Supplementary Methods. [file 12885_2021_7806_MOESM1_ESM.docx]

**Additional file 1:**

**Supplementary Methods**

**Validation of BCOR mutation by Sanger sequencing**

The following primers were used for PCR amplification and Sanger Sequencing. E197X forward 5’-GGTCAATCCTTACATGTAGGGTGCCACGCCAG-3’ and E197X reverse 5’- CTGGCGTGGCACCCTACATGTAAGGATTGACC-3’. W289X forward 5’-GACAAAAGCCTCCCGTAGAAGATGGGCGTCAG-3’ and W289X reverse 5’- CTGACGCCCATCTTCTACGGGAGGCTTTTGTC-3’.

**Antibodies and immunoblotting**

Cells were lysed in RIPA buffer (50 mM Tris-Cl, pH 7.4, 150 mM NaCl, 0.1% NaN_3_, 1% Nonidet P-40, 0.25% sodium deoxycholate, 1 mM EDTA, 1 mM Na_3_VO_4_, 1 mM NaF, and protease inhibitor cocktail) for 30 min on ice. Proteins were then quantified using BCA assay kit (Pierce). Cell lysates were resolved by SDS–PAGE and transferred to PVDF membranes. Membranes were blocked with 5% non-fat dry milk and probed with anti-FLAG (Sigma), anti-BCOR (Bethyl Laboratoris), anti-BCL6 (Cell Signaling), anti-PCGF1 (Abcam), anti-RING1B (Cell Signaling), and anti-phospho-AKT (Ser473) (Cell Signaling) antibodies followed by probing with horseradish peroxidase-conjugated secondary antibody (Bio-Rad). Anti-AKT (Cell Signaling) and α-tubulin (Santa Cruz Biotechnology) antibodies were used as loading controls. Immunostained proteins were detected using an ECL detection system (Amersham Pharmacia Biotech). All western blot experiments were performed in at least three independent experiments and representative images from one experiment have been presented.

**Cell proliferation and cytokine assays**

At 48 hours after transfection, Jurkat (human T cell acute lymphoblastic leukemia) and Hut-78 (human cutaneous T-cell lymphoma) cells were seeded in 96-well plates in triplicate at a density of 5 ×10^3^ cells/well in 100 μL of RPMI-1640 medium containing 10% FBS and antibiotics. For stimulation, a 96-well plate was coated with 10 μg/mL of anti-CD3 (HIT3a, BD Pharmingen) and 2 μg/mL of anti-CD28 (CD28.2 BD Pharmingen) overnight at 4°C. Cell proliferation was evaluated using Cell Counting Kit-8 (Dojindo) according to the manufacturer's instructions, and the absorbance value for each well was measured at 450 nm using a microplate reader (Spectra Max 180, Molecular Devices). Each experiment was repeated three times. For cytokine assays, cells were stimulated with 15 ng/mL phorbol myristate acetate (PMA) and 290 ng/mL ionomycin (eBioscience) for 4 hours. After 24 hours, the supernatants were examined using Human IL-2 ELISA kits (Thermo Scientific) according to the manufacturer's instructions. Each experiment was repeated three times.
