# Supplementary material for: The mutation of BCOR is highly recurrent and oncogenic in mature T-cell lymphoma
Source: BMC Cancer. 2021 Jan 19;21:82. doi: 10.1186/s12885-021-07806-8 (PMC7816311; doi:10.1186/s12885-021-07806-8)
Supplement: Supplementary file 2 — Additional file 2: Figure S1. Validation of K607E mutation on BCOR in FFPE tissues and fresh frozen tissues from the same patients. Representative sequencing traces of wild-type and K607E mutant BCOR. Lysine (AAG) was changed to Glutamic acid (GAG) at the 607th amino acid of BCOR. Arrows and red letters denote the location of the base change (#1–5 wild-type BCOR samples and #6–10 BCOR K607E mutant samples). [file 12885_2021_7806_MOESM2_ESM.docx]

**Additional file 2:**


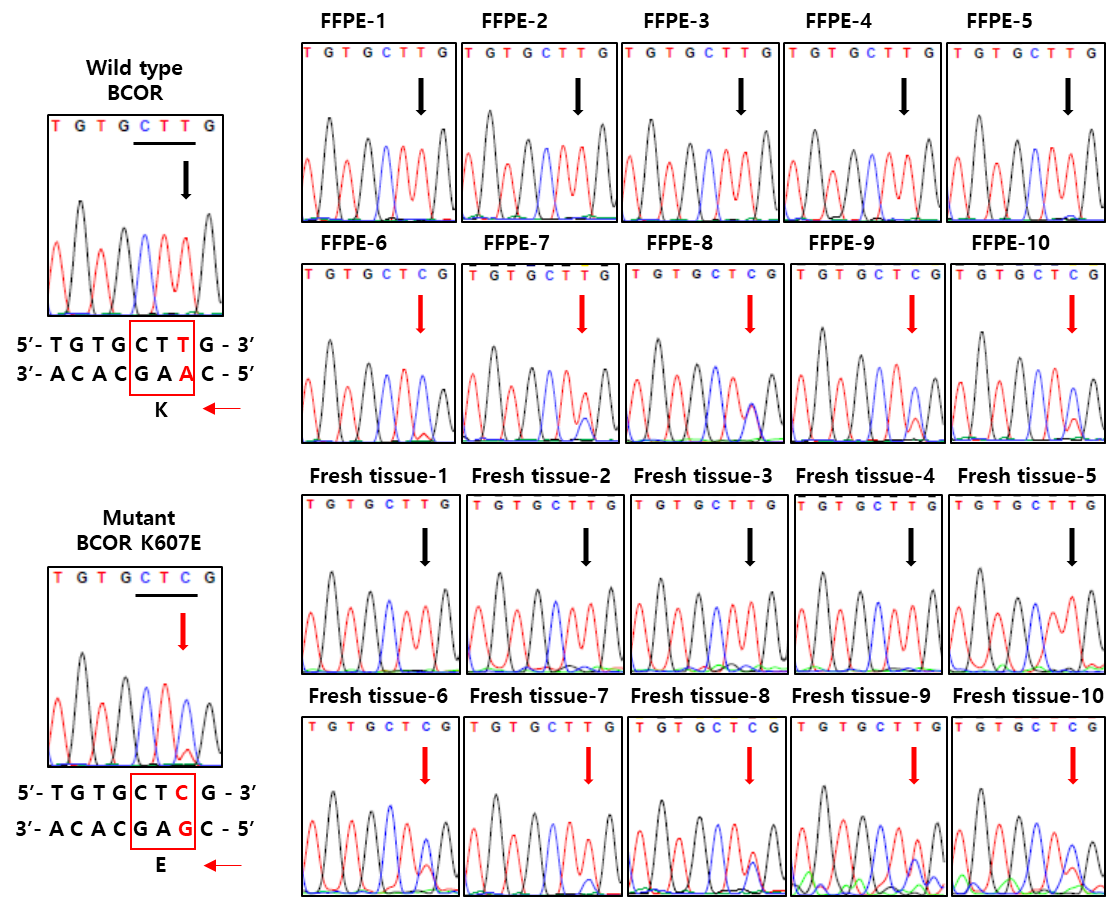
**Supplementary Figure S1**

**Figure S1.** Validation of K607E mutation on BCOR in FFPE tissues and fresh frozen tissues from the same patients. Representative sequencing traces of wild-type and K607E mutant BCOR. Lysine (AAG) was changed to Glutamic acid (GAG) at the 607th amino acid of BCOR. Arrows and red letters denote the location of the base change (#1-5 wild-type BCOR samples and #6-10 BCOR K607E mutant samples).
