# Supplementary material for: The mutation of BCOR is highly recurrent and oncogenic in mature T-cell lymphoma
Source: BMC Cancer. 2021 Jan 19;21:82. doi: 10.1186/s12885-021-07806-8 (PMC7816311; doi:10.1186/s12885-021-07806-8)
Supplement: Supplementary file 3 — Additional file 3: Figure S2. Expression of BCOR K607E mutant enhanced cell proliferation and IL- 2 production. a Hut78 cells were transfected with wild-type BCOR or K607E mutant expressing plasmids. After 48 h, cells were stimulated with plate-bound anti-CD3/CD28. After stimulation, cell proliferation was determined using a cell counting kit (CCK-8). Data are shown as the mean ± SEM of seven independent experiments performed in triplicate (**P < 0.01 compared with cells expressing wild-type BCOR). b After transfection, Hut78 cells were stimulated with PMA and ionomycin. The concentrations of IL-2 were estimated by ELISA. Data are shown as the mean ± SEM of five independent experiments performed in triplicate (**P < 0.01 compared with cells expressing wild-type BCOR). [file 12885_2021_7806_MOESM3_ESM.docx]

**Additional file 3:**

**Supplementary Figure S2**

**
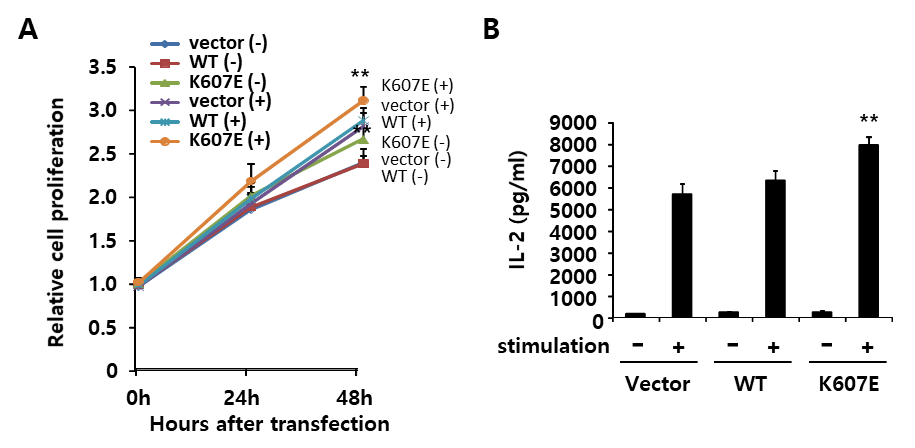
**

**Figure S2.** Expression of BCOR K607E mutant enhanced cell proliferation and IL- 2 production. **a** Hut78 cells were transfected with wild-type BCOR or K607E mutant expressing plasmids. After 48 hours, cells were stimulated with plate-bound anti-CD3/CD28. After stimulation, cell proliferation was determined using a cell counting kit (CCK-8). Data are shown as the mean ± SEM of seven independent experiments performed in triplicate (**P<0.01 compared with cells expressing wild-type BCOR). **b** After transfection, Hut78 cells were stimulated with PMA and ionomycin. The concentrations of IL-2 were estimated by ELISA. Data are shown as the mean ± SEM of five independent experiments performed in triplicate (**P<0.01 compared with cells expressing wild-type BCOR).
