# Supplementary material for: The mutation of BCOR is highly recurrent and oncogenic in mature T-cell lymphoma
Source: BMC Cancer. 2021 Jan 19;21:82. doi: 10.1186/s12885-021-07806-8 (PMC7816311; doi:10.1186/s12885-021-07806-8)
Supplement: Supplementary file 4 — Additional file 4: Figure S3. HOXB6, ISPD, ATP13A4, MAK, and SLC7A8 were up-regulated in BCOR K607E mutant expressing cells (a) and BCOR K607E mutant tumor samples (b). Quantitative PCR analysis of the indicated genes from the list of Top10 genes showed upregulation by BCOR K607E mutant. GAPDH was used as a control to normalize the levels of these transcripts. Data are shown as the mean ± SEM of six independent experiments performed in triplicates (*P < 0.05, **P < 0.01 compared with cells transfected with wild-type BCOR and wild-type BCOR tumor samples). [file 12885_2021_7806_MOESM4_ESM.docx]

**Additional file 4:**

**Supplementary Figure S3**


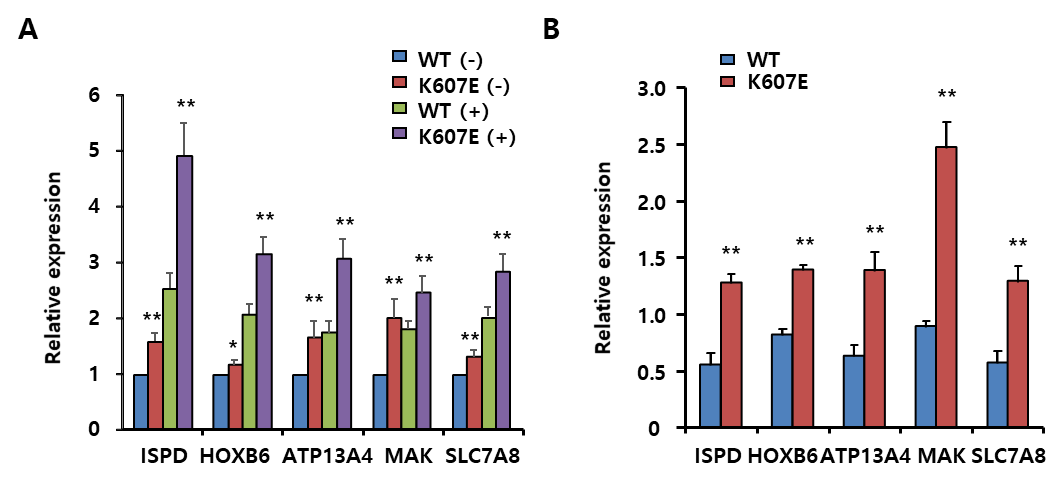


**Figure S3.** HOXB6, ISPD, ATP13A4, MAK, and SLC7A8 were up-regulated in BCOR K607E mutant expressing cells (a) and BCOR K607E mutant tumor samples (b). Quantitative PCR analysis of the indicated genes from the list of Top10 genes showed upregulation by BCOR K607E mutant. GAPDH was used as a control to normalize the levels of these transcripts. Data are shown as the mean ± SEM of six independent experiments performed in triplicates (*P<0.05, **P<0.01 compared with cells transfected with wild-type BCOR and wild-type BCOR tumor samples).
