# Supplementary figures and images for: The mutation of BCOR is highly recurrent and oncogenic in mature T-cell lymphoma
Source: BMC Cancer. 2021 Jan 19;21:82. doi: 10.1186/s12885-021-07806-8 (PMC7816311; doi:10.1186/s12885-021-07806-8)

**Additional file 5:**

**Supplementary Figure S4. The uncropped blots of Fig 2A and 2C are displayed.**


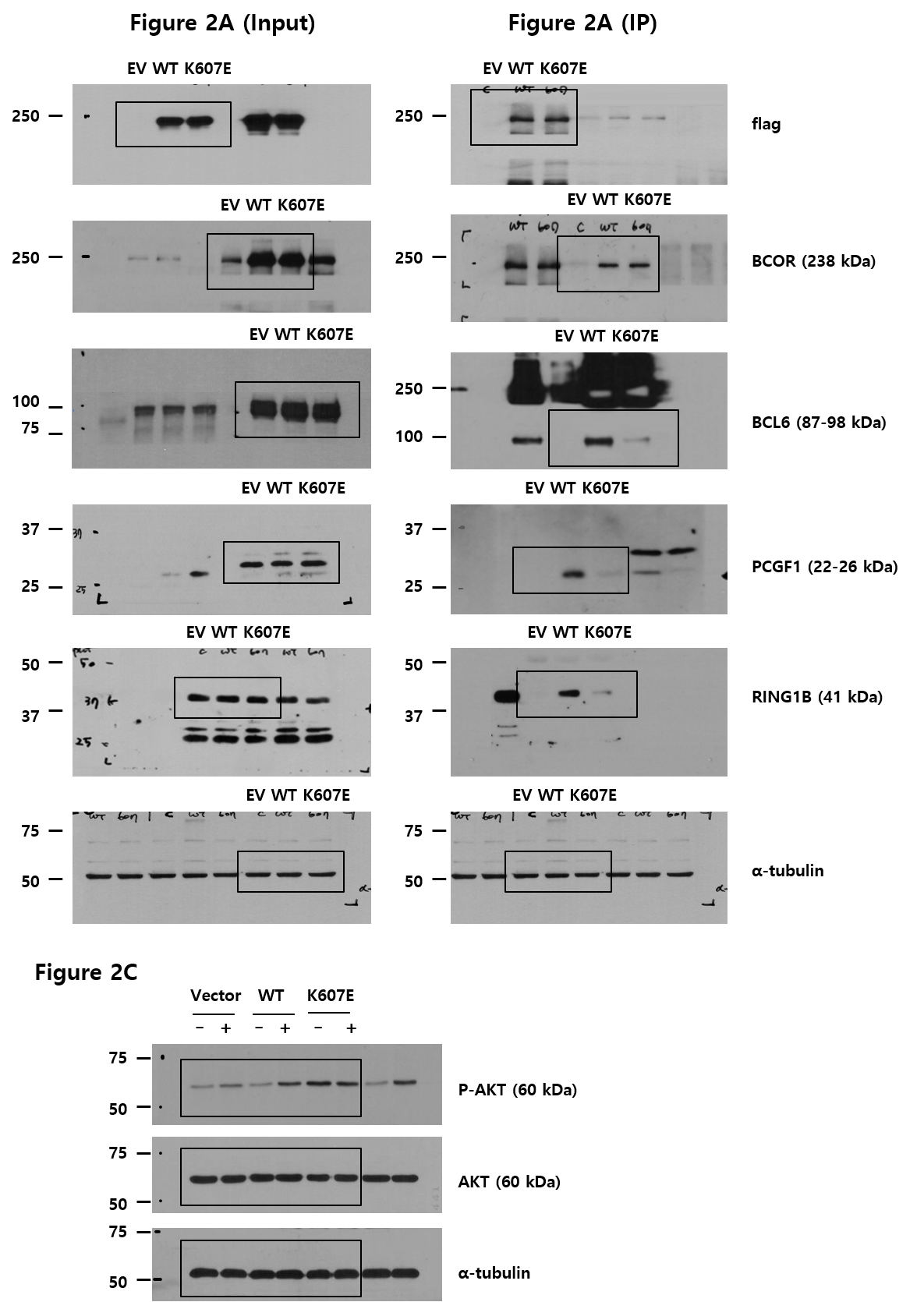

Supplement: Supplementary file 5 — Additional file 5: Figure S4. The uncropped blots of Fig. 2a and c are displayed. [file 12885_2021_7806_MOESM5_ESM.docx]

**Additional file 6:**

**Supplementary Figure S5. The uncropped blots of Fig 3 are displayed.**


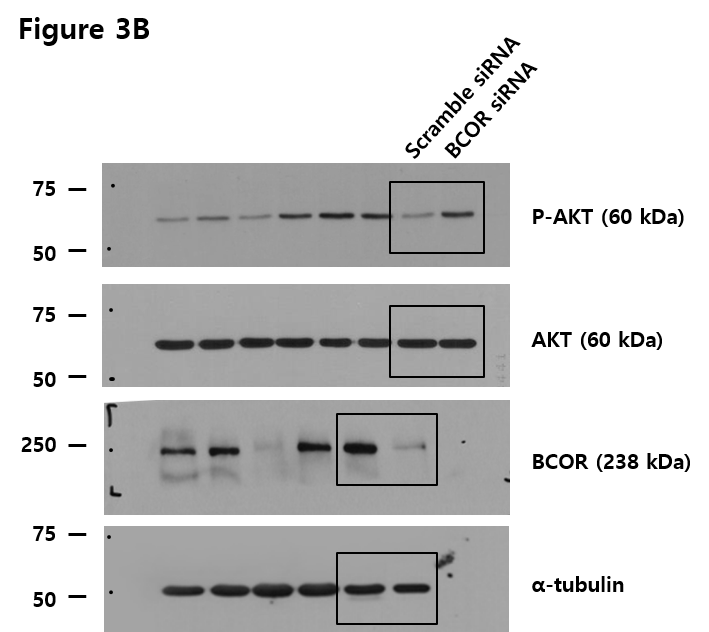

Supplement: Supplementary file 6 — Additional file 6: Figure S5. The uncropped blots of Fig. 3b are displayed. [file 12885_2021_7806_MOESM6_ESM.docx]
